# Supplementary material for: Adaptive Sparse Multi-Block PLS Discriminant Analysis: An Integrative Method for Identifying Key Biomarkers from Multi-Omics Data
Source: Genes (Basel). 2023 Apr 23;14(5):961. doi: 10.3390/genes14050961 (PMC10218045; doi:10.3390/genes14050961)
Supplement: Supplementary file 1 [file genes-14-00961-s001.zip › genes-2281029-supplementary.docx]

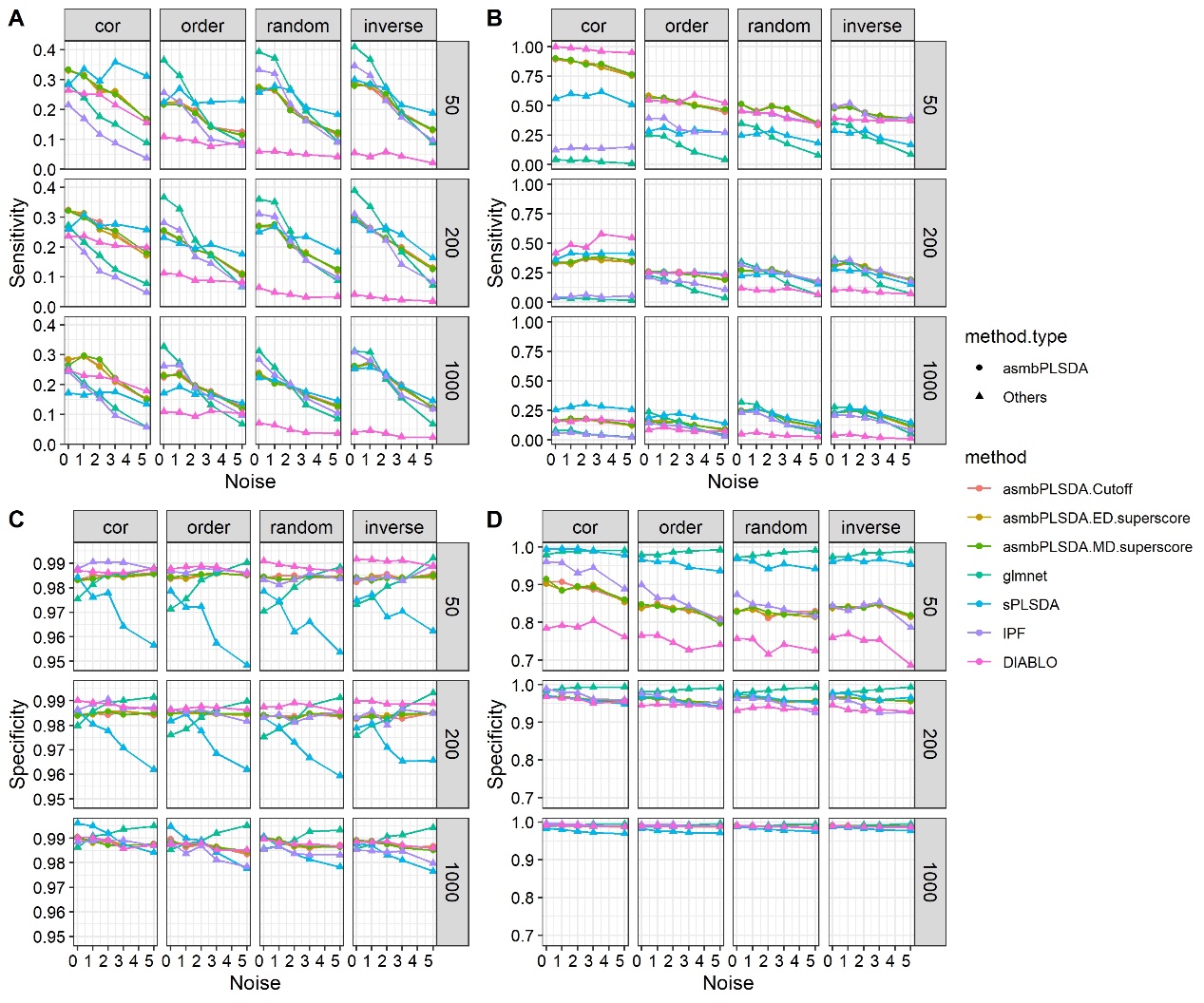


**Figure S1.** The sensitivity and specificity of different methods for different blocks for simulation data with the binary outcome.


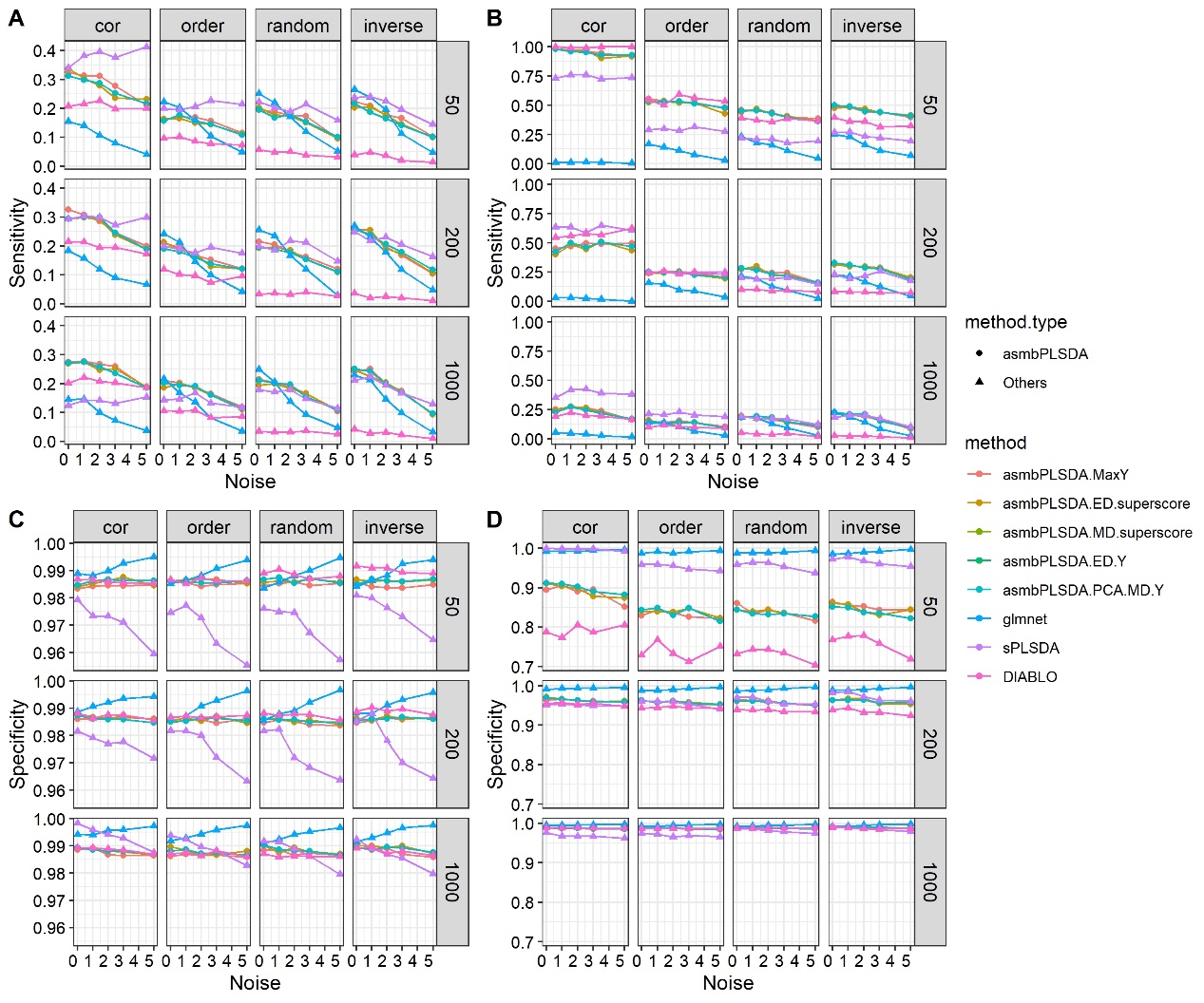


**Figure S2.** The sensitivity and specificity of different methods for different blocks for simulation data with the multiclass outcome.

**
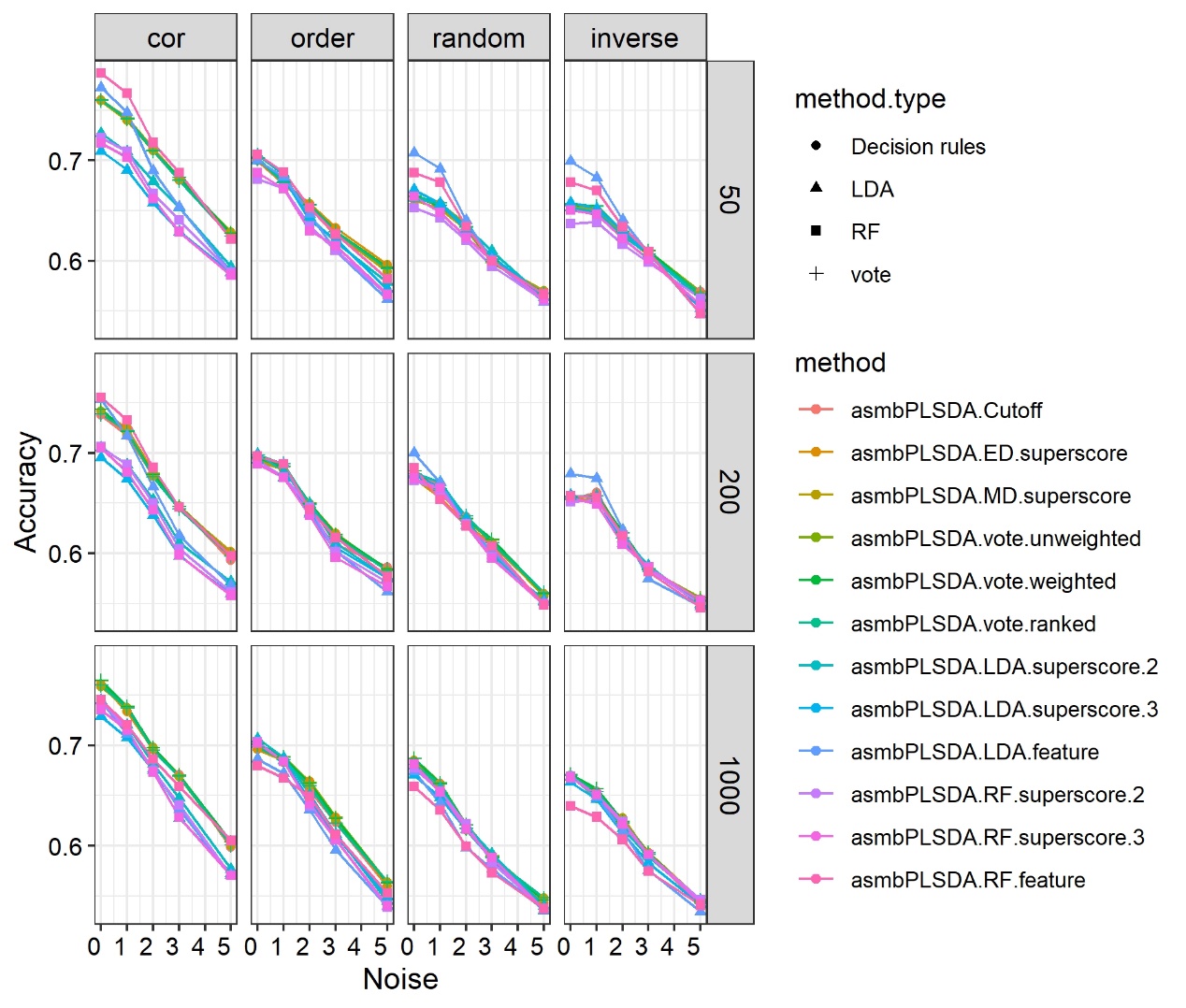
Figure S3**. The classification results for simulation data with the binary outcome among asmbPLS-DA methods.

**
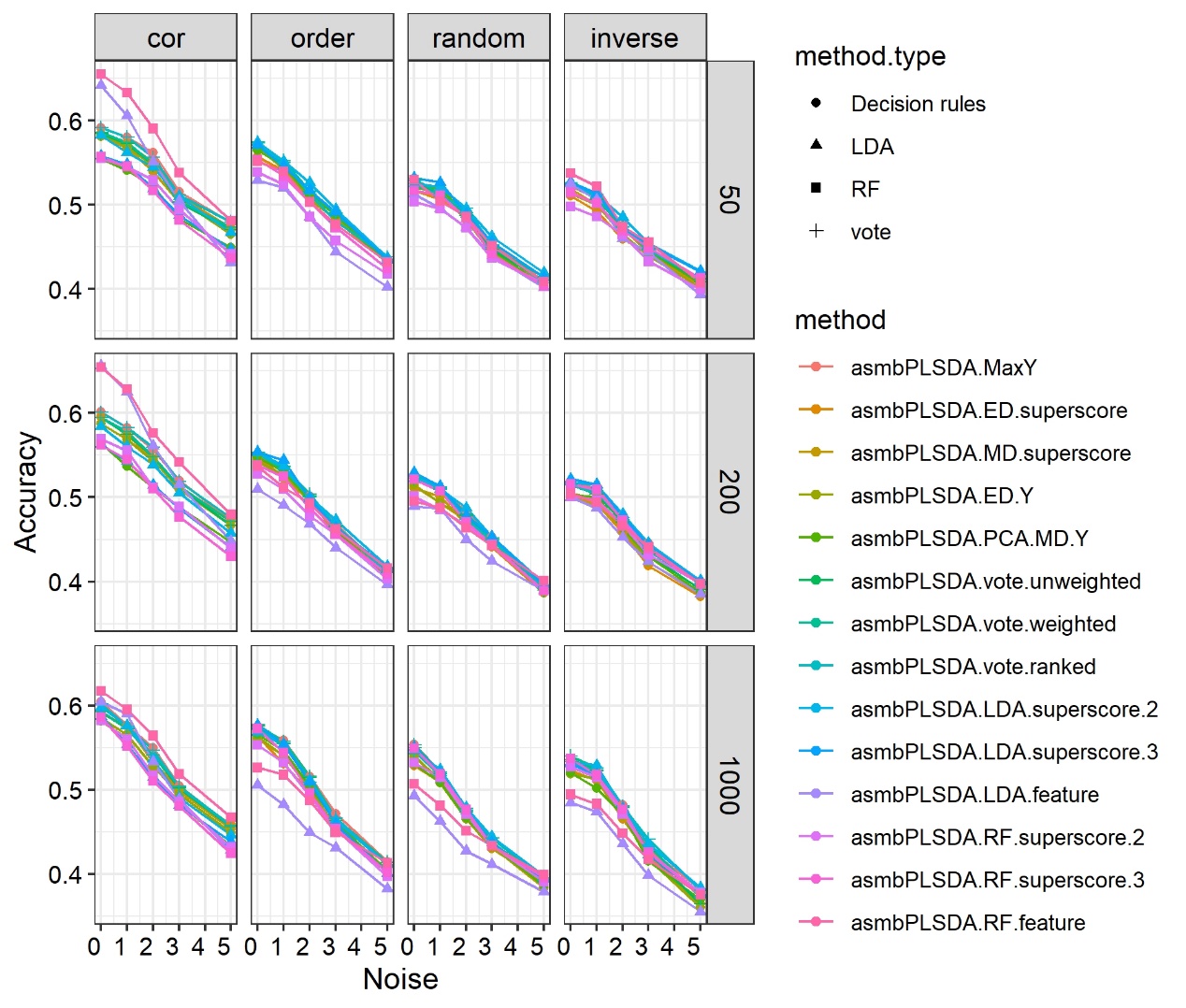
Figure S4**. The classification results for simulation data with the multiclass outcome among asmbPLS-DA methods.

**Table S1.** Comparison of the classification performance for different methods using the real data with additional protein block included and the mutliclass outcome.

| Method | Overall accuracy | Recall for Stage I group | Recall for Stage II group | Recall for Stage III + Stage IV group | Balanced accuracy |
| --- | --- | --- | --- | --- | --- |
| asmbPLS-DA with Max Y | 0.4331 | 0.4160 | 0.5229 | 0.2442 | 0.3944 |
| asmbPLS-DA with LDA | 0.5657 | 0.2320 | 0.8438 | 0.1429 | 0.4062 |
| asmbPLS-DA with RF | 0.5827 | 0.0000 | 0.9667 | 0.0691 | 0.3453 |
| sPLS-DA | 0.5341 | 0.0000 | 0.8417 | 0.1613 | 0.3343 |
| L1-regularized multinomial logistic regression | 0.5803 | 0.0000 | 0.9917 | 0.0046 | 0.3321 |
